# Host metabolism shapes the intestinal microbiota: a top-down paradigm of environmental selection pressure

**DOI:** 10.1080/19490976.2026.2667735

**Published:** 2026-05-07

**Authors:** Ziyu Ma, Huairuo Shi, Xinjie Bai, Zixu Wang, Jing Cao, Yulan Dong, Yaoxing Chen

**Affiliations:** a State Key Laboratory of Veterinary Public Health and Safety, College of Veterinary Medicine, China Agricultural University, Haidian, Beijing, People's Republic of China; b College of Animal Science and Technology, Beijing Vocational College of Agriculture, Fangshan, Beijing, People's Republic of China

**Keywords:** Facultative, obligate anaerobes, top-down regulation, intestinal dysbiosis, Mitochondrial β-oxidation, physiological hypoxia, nutritional immunity

## Abstract

Intestinal homeostasis is not a stochastic microbial assembly but a deterministic outcome orchestrated by host-mediated metabolic gating. Traditional research has prioritized the microbiota’s impact on host physiology. However, the consistent expansion of facultative anaerobes, such as *Enterobacteriaceae*, observed in pathological states like intestinal inflammation, suggests that dysbiosis is fundamentally a consequence of impaired host regulation. Here, we propose a “top-down” paradigm of host metabolic regulation, framing the host as an “ecological engineer” that actively shapes the microbiome through metabolism. We detail three critical metabolic filters: (1) the maintenance of epithelial hypoxia via mitochondrial *β*-oxidation to suppress aerobic respiration; (2) the implementation of “nutritional immunity” to restrict glucose and inflammation-derived electron acceptors (nitrate and tetrathionate); and (3) the energy-dependent synthesis of the gel-forming mucin 2 (*MUC2*) mucus layer and antimicrobial peptides (AMPs). We argue that the breakdown of these filters leads to “niche opening,” which acts as the fundamental driver of dysbiosis. Finally, we discuss therapeutic strategies aimed at restoring host bioenergetics—including Peroxisome proliferator-activated receptor gamma (*PPAR-γ*) agonists, melatonin, and ketogenic diets—to rebuild the host's ecological filtration capacity and fundamentally correct dysbiosis.

## 1. Introduction

The mammalian intestinal ecosystem is defined by a strictly anaerobic microenvironment, which determines the symbiotic dominance of obligate anaerobes from the phyla *Bacillota* (formerly *Firmicutes*) and *Bacteroidota* (formerly *Bacteroidetes*).^1^ This relationship forms the foundation of host metabolic and immune homeostasis.^2‐4^ Concurrently, facultative anaerobes, particularly the family *Enterobacteriaceae*, are strictly confined to low abundances.^5‐7^ Once the balance between obligate and facultative anaerobes is disrupted, a downward spiral is triggered. Beyond metabolic competition, proliferating pathogens release pro-inflammatory ligands and virulence factors—such as hemolysins and lipopolysaccharide (LPS)—causing tissue damage. These microbial insults exacerbate bioenergetic depletion, trapping the host in a state of persistent chronic inflammation.^8^

Historically, research has predominantly adopted a “bottom-up” perspective,^9^ emphasizing how microbial metabolites Short-Chain Fatty Acids (SCFAs), bile acids, tryptophan and LPS regulate host physiology.^10^^,^^11^ However, this unidirectional approach fails to account for why dysbiosis—specifically the expansion of facultative anaerobes—exhibits such striking consistency across diverse pathologies like inflammatory bowel disease (IBD), diabetes and obesity.^12‐14^ This conserved phenotype challenges the notion of stochastic dysbiosis, strongly suggesting that host metabolic alterations exert a unified “environmental selection pressure” on microbial composition.^15^^,^^16^ We contend that metabolic dysfunction caused by host disease resets the microbial habitat, shifting the intestinal fermentation metabolism from favoring strict anaerobes to promoting the respiratory growth of facultative anaerobes fueled by oxygen and nitrate, thereby triggering dysbiosis.^17^. Therefore, understanding how host metabolic alterations reshape this ability to select intestinal microbiota is crucial for regulating microbial composition.^18^

Here, we propose a “top-down” regulatory paradigm: the host acts not as a passive vessel for microbes but as an “ecological engineer” that actively shapes the microbiome structure via bioenergetics. Specifically, we argue that the host constructs “ecological filters” through three key metabolic mechanisms: (1) the maintenance of epithelial hypoxia via mitochondrial *β*-oxidation to inhibit the respiration of facultative anaerobes; (2) the implementation of “nutritional immunity” to restrict the bioavailability of glucose and electron acceptors (nitrate); and (3) the reliance on high-energy metabolism to sustain the Mucin 2, oligomeric mucus/gel-forming (*MUC2*) mucus and antimicrobial peptide barriers. We posit that the “niche opening” resulting from host metabolic dysregulation is the fundamental driver of dysbiosis. This review details these mechanisms and explores intervention strategies aimed at restoring host bioenergetics—such as roxisome proliferator-activated receptor gamma (*PPAR-γ*) agonizts, melatonin and ketogenic diets (KD)—to correct intestinal dysbiosis at its root by rebuilding the host's “ecological filtering” capacity.

## 2. Metabolic dynamics of the intestinal microbiota: fermentation core and respiratory expansion

**Figure 1.**
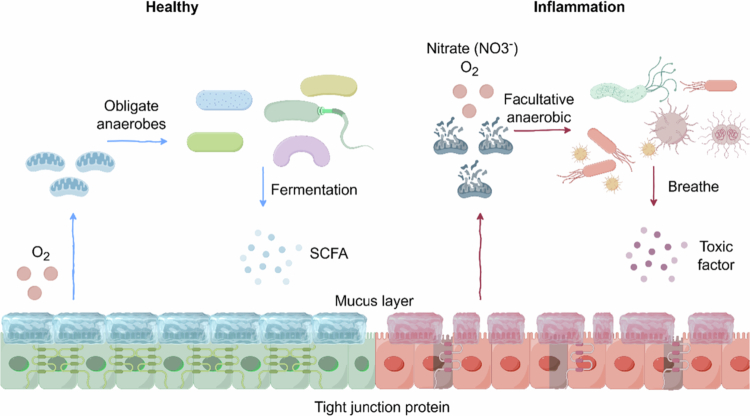
Host-mediated microbial filtration mechanisms determine intestinal microbiota homeostasis and dysbiosis. Under healthy conditions, host intestinal epithelial cells efficiently metabolize Short-Chain Fatty Acids (SCFAs) via mitochondrial *β*-oxidation, maintaining the physiological hypoxic environment in the intestinal lumen. This hypoxic environment, combined with the protective, thick, mucus/gel-forming (MUC2) layer and antimicrobial peptides (AMPs), forms a stringent “microbiota filter.” This environment selectively favors obligate anaerobes as the dominant microbiota, which ferment dietary fiber to produce short-chain fatty acids, thereby benefiting the host. During inflammation or metabolic stress, host mitochondria become impaired. Epithelial cells switch to glycolytic metabolism, causing oxygen leakage into the intestinal lumen. Concurrently, inflammation-induced reactive oxygen species (ROS) react with nitric oxide (NO) to form nitrates. This influx of high-energy electron acceptors triggers an explosion of facultative anaerobic bacteria, whose growth rate overwhelms that of obligate anaerobes. The expansion of facultative anaerobes releases virulence factors, accelerating microbial ecosystem collapse. Created in Figdraw.com.

Intestinal microbiota homeostasis is defined not merely by species composition but by a dynamic equilibrium between distinct metabolic modes. Understanding this balance hinges on deciphering how two fundamental energy acquisition pathways—“fermentation” and “respiration”—dictate competitive advantages within the intestinal niche (Figure 1).

### 2.1. The fermentation lifestyle of obligate anaerobes

The cornerstone of the intestinal ecosystem is the “fermentation core,” dominated by obligate anaerobes from the phyla *Bacteroidota* and *Bacillota*. Adapted to the strictly hypoxic environment of the colon, these microbes must rely on substrate-level phosphorylation for energy production in the absence of exogenous high-potential electron acceptors, such as oxygen or nitrate.^1^ To compensate for the low ATP yield of fermentation, these microbes have evolved a “low-efficiency, high-throughput” survival strategy: rapidly consuming substrates while excreting large quantities of SCFAs.^19^^,^^20^ This strategy has profound ecological implications. It not only elevates luminal SCFAs concentrations to fuel the host but also rapidly depletes soluble carbohydrates, establishing “colonization resistance” via nutrient deprivation against invasive pathogens.^21^^,^^22^

Notably, while some obligate anaerobes (*Bacteroides fragilis*) possess a limited capacity for heme-dependent fumarate respiration under specific conditions,^23^ this ability is severely constrained. Crucially, key fermentation enzymes, such as glycyl radical enzymes, are highly oxygen-sensitive and undergo irreversible inactivation upon exposure.^24^ This molecular sensitivity fundamentally confines these taxa to the strictly anoxic colonic lumen (Figure 1).

### 2.2. The respiratory potential of facultative anaerobes

In contrast to the homeostatic obligate anaerobes, facultative anaerobes—typified by the family *Enterobacteriaceae*—act as “opportunists” equipped with versatile metabolic genomes that encode complete electron transport chains. In a healthy, hypoxic intestine, the scarcity of electron acceptors forces the bacteria into inefficient fermentation, thereby suppressing their population growth.^25^^,^^26^ However, the appearance of high-potential alternative electron acceptors (such as inflammation-derived nitrate or tetrathionate) allows these bacteria to rapidly reorganize their electron transport chains. By leveraging efficient respiratory metabolism, they gain a significant growth advantage.^27^^,^^28^ For instance, *Salmonella* can exploit host-driven inflammation to oxidize thiosulfate into tetrathionate for respiratory utilization, thereby outcompeting resident microbes.^29^

Fundamentally, intestinal homeostasis stems from the host's active filtering of electron acceptors, such as oxygen and nitrate. Once inflammation triggers an influx of these acceptors, facultative anaerobes leverage their “respiratory advantage” to outcompete fermentative bacteria by producing more ATP and biomass, ultimately disrupting homeostasis.^30^^,^^31^ Consequently, the host's maintenance of “physiological hypoxia” and nutrient restriction is the linchpin of this ecological filter—a mechanism explored in depth in the following sections (Figure 1). In conclusion, the competitive hierarchy of the intestinal microbiome is fundamentally dictated by bioenergetics. While obligate anaerobes dominate the healthy intestine via “high-throughput fermentation,” this dominance is ecologically fragile and strictly contingent upon an electron-acceptor-limited environment. The influx of oxygen or nitrate instantly shifts the metabolic advantage in favor of facultative anaerobes, driving dysbiosis through “respiratory expansion.” Thus, the preservation of intestinal homeostasis depends less on the microbes themselves and more on the host’s ability to rigorously enforce a hypoxic niche.^15^^,^^32^ It is important to clarify that while obligate anaerobes dominate the healthy intestine, the fermentation core is not composed solely of beneficial symbionts. Certain obligate anaerobic pathogens, such as *Clostridioides difficile* and *Fusobacterium nucleatum*, can exploit specific ecological niches to cause disease.^33^^,^^34^ This indicates that the host's ecological filtering mechanism is determined not by taxonomic identity, but by metabolic patterns. Top-down pressures primarily influence energy-acquisition patterns: they favor fermentative microbial communities while actively excluding respiratory, opportunistic bacteria. Thus, the expansion of *Clostridioides difficile* in colorectal cancer, for instance, reflects the opening of ecological niches where pathogenic symbionts circumvent standard filtering mechanisms by exploiting abnormal peptide metabolism or necrotic byproducts—rather than relying on the oxygen-driven respiratory expansion typical of *Enterobacteriaceae.*^35^

## 3. Host mechanisms of intestinal oxygen metabolism

The efficacy of host metabolic regulation of the microbiota is strictly determined by the gastrointestinal tract's anatomical geography. In the small intestine, characterized by high material transit rates and relatively elevated oxygen concentrations, the host primarily relies on nutritional immunity (such as glucose sequestration) and chemically gated mechanisms mediated by antimicrobial peptides derived from Paneth cells.^36^^,^^37^ Conversely, the distal colon becomes the dominant force through an ultra-anaerobic environment sustained by mitochondrial *β*-oxidation.^38^ This spatial gradient reinforces oxygen-consuming zones in areas with the highest density of fermentative symbionts, thereby preventing pathogens from invading the microbial core through respiration.^39^

Despite the rich vascular supply in the mammalian submucosa, the healthy colonic lumen maintains a state of strict “physiological hypoxia”.^40^ This steep oxygen gradient is not a passive physical barrier but the result of active metabolism by host epithelial cells, constituting the first and most critical ecological filter that selects for obligate anaerobes while suppressing facultative anaerobes.^39^ Physiologically, the capillary network in the lamina propria maintains an oxygen partial pressure (pO₂) of approximately 40–50 mmHg. As oxygen diffuses apically, robust epithelial oxygen consumption acts as a metabolic sink, dropping intracellular pO₂ to 5–10 mmHg. This continuous depletion ensures the healthy colonic lumen remains profoundly hypoxic, with a pO₂ consistently below 1–3 mmHg.^41^^,^^42^ When this epithelial filter fails, the gradient collapses. The resulting leakage of unconsumed oxygen into the lumen elevates local pO₂ beyond the physiological tolerance threshold of obligate anaerobes (like  >  10 mmHg), thereby opening a respiratory niche that favors the expansion of facultative anaerobes.^43^^,^^44^

Differentiated colonic epithelial cells possess a unique metabolic profile: unlike most glucose-dependent somatic cells, they prioritize microbial fermentation products—specifically SCFAs, such as butyrate—as their primary fuel source.^45^ Upon cellular entry, butyrate undergoes highly efficient mitochondrial *β*-oxidation and tricarboxylic acid (TCA) cycle metabolism, a process that requires substantial oxygen consumption.^46^ Given the high capacity of colonocytes for butyrate uptake, this pathway effectively transforms the epithelium into a massive “bioenergetic oxygen sink,” depleting oxygen as it diffuses from the vasculature toward the lumen. The efficacy of this metabolic filter exhibits pronounced regional heterogeneity along the colonic axis. Dietary fiber fermentation predominantly occurs in the proximal colon, yielding peak luminal concentrations of SCFAs, including butyrate, in the cecum and the ascending colon. When supplied with abundant substrate, proximal colonocytes sustain high rates of *PPAR-γ*-driven *β*-oxidation, serving as a robust primary oxygen sink. In contrast, as digesta travels distally, fermentable carbohydrates are progressively depleted, leading to a physiological decline in butyrate availability.^47^^,^^48^ The physiological proximal-to-distal depletion of luminal SCFAs leaves distal colonocytes operating on a fragile bioenergetic margin. This localized butyrate deficiency predisposes the distal epithelial oxygen sink to collapse under metabolic stress, which may explain the characteristic onset of ulcerative colitis in this region.^49^^,^^50^

This high-demand oxygen state is tightly governed by the nuclear receptor *PPAR-γ*. Acting as an intracellular “bacterial sensor,” *PPAR-γ* is activated by butyrate to drive the transcription of *β*-oxidation genes Acyl-CoA dehydrogenase short chain (*Acads*), Carnitine palmitoyltransferase 1 A (*Cpt1a*) and key regulators of mitochondrial biogenesis Peroxisome proliferator-activated receptor gamma coactivator 1-alpha (*PGC-1α*), Nuclear respiratory factor 1 (*NRF*), Mitochondrial transcription factor A (*TFAM*), which in turn promote the expression of oxidative phosphorylation subunits such as Succinate dehydrogenase complex iron sulfur subunit B (*SDHB*) and Cytochrome c oxidase subunit 4I1(*COX4I1*).^51‐53^ This establishes a homeostatic host-microbiota feedback loop: obligate anaerobes ferment fiber to produce butyrate; the host consumes oxygen to metabolize that butyrate; and the resulting hypoxia protects obligate anaerobes from oxygen toxicity while stabilizing host Hypoxia-Inducible Factor (*HIF-1α*) to reinforce the barrier.^32^^,^^54^ Conversely, downregulation of *PPAR-γ* activity attenuates epithelial *β*-oxidation, leading to luminal “oxygenation” that precipitates the loss of obligate anaerobes and the secondary expansion of facultative anaerobes.^55^^,^^56^

**Figure 2.**
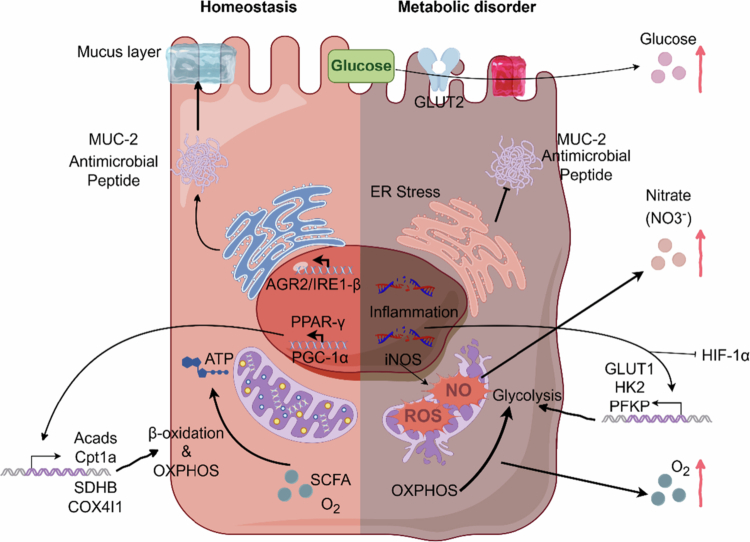
Host metabolic reprogramming determines selectivity for intestinal microbiota. 1. Left Side (Homeostatic Pathway): In the cell nucleus, Peroxisome proliferator-activated receptor gamma (*PPAR-γ*) is activated by microbial metabolites (SCFAs) and recruits the transcriptional coactivator *PGC-1α*. This complex drives the transcription of genes involved in mitochondrial *β*-oxidation, like Acyl-CoA dehydrogenase short chain (Acads), Carnitine palmitoyltransferase 1 A (Cpt1a), and oxidative phosphorylation (OXPHOS) subunits, Succinate dehydrogenase complex iron sulfur subunit B (*SDHB*), Cytochrome c oxidase subunit 4I1(*COX4I1*). The coupling of fatty acid *β*-oxidation with efficient OXPHOS ensures high oxygen consumption by the epithelium, thereby maintaining physiological hypoxia in the intestinal lumen. Concurrently, the endoplasmic reticulum, monitored by the UPR pathway Inositol-requiring enzyme 1 beta (*IRE1β*)/Anterior gradient 2 (AGR2), efficiently folds and secretes MUC2 mucin and antimicrobial peptides, thereby preserving the intestinal barrier. 2. Right side (pathological pathway): Inflammation suppresses *PPAR-γ* signaling while stabilizing Hypoxia-inducible factor 1-alpha (*HIF-1α*). This transcriptional switch forces a metabolic shift from mitochondrial oxidative phosphorylation to aerobic glycolysis (the Warburg effect). upregulates glycolytic genes Glucose Transporter 1 (*GLUT1*), Hexokinase 2 (*HK2*), and Phosphofructokinase, Platelet (*PFKP*), thereby reducing epithelial oxygen consumption and causing oxygen leakage into the lumen. Concurrently, severe endoplasmic reticulum (ER) stress disrupts the protein-folding machinery (blocking the IRE1β/AGR2 axis) and impairs *MUC2* and antimicrobial peptide secretion. Furthermore, inflammatory mediators induce inducible nitric oxide synthase (*iNOS*) to produce NO, which generates nitrate (an electron acceptor) in the presence of ROS, while the loss of GLUT2 polarity causes glucose leakage (a carbon source), collectively creating a niche for facultative anaerobes. Created in Figdraw.com.

However, during intestinal inflammation, infection, or severe metabolic stress, this metabolic switch is flipped. Epithelial metabolism shifts abruptly from efficient oxidative phosphorylation to inefficient anaerobic glycolysis (close to the Warburg effect), a transition marked by upregulation of glycolytic genes such as Glucose Transporter 1 (GLUT1), Hexokinase 2 (HK2), and Platelet Phosphofructokinase (*PFKP*).^57‐59^ This metabolic reprogramming floods the lumen with oxygen. Furthermore, reactive oxygen species (ROS) and nitric oxide (NO) released during the immune response directly target mitochondrial metabolic nodes: ROS disrupt the iron-sulfur clusters of aconitase, blocking the TCA cycle,^60^^,^^61^ while NO competitively inhibits cytochrome *c* oxidase, stifling mitochondrial respiration.^62^ This mitochondrial dysfunction causes uncontrolled oxygen leakage into the lumen, driving a vicious “inflammation-metabolism-barrier” cycle. Luminal oxygenation activates respiratory pathways in facultative anaerobes (*Enterobacteriaceae*), enabling them to outcompete obligate anaerobes via superior energy yields and exponential growth.^15^ For instance, increased intestinal oxygenation in colorectal cancer patients drives the proliferation of toxin-producing *Escherichia coli,*^63^ and mitochondrial dysfunction in irritable bowel syndrome (IBS) is closely linked to an abundance of facultative anaerobes such as *Parastaphylococcus* and *Haemophilus parainfluenzae.*^64^^,^^65^ In essence, the epithelium's “oxygen sink” function serves as the primary metabolic checkpoint governing microbial composition. The active maintenance of hypoxia is not merely a byproduct of cellular respiration but a deliberate ecological filter that segregates host tissue from the anaerobic lumen. When this bioenergetic barrier collapses due to metabolic reprogramming, oxygen leakage provides the initial spark for the expansion of facultative anaerobes.^42^ However, oxygen is not the sole currency in this competitive economy; the host also regulates the availability of critical substrates such as carbon sources and electron acceptors (Figure 2).

## 4. Host nutrient metabolism and microbial resource competition

The intestinal lumen is not a nutrient-replete sanctuary for bacteria but a fiercely competitive ecological niche characterized by the scarcity of carbon, nitrogen, and trace elements. To limit the growth of potential pathogens, the host employs a defense strategy known as “nutritional immunity.” Although nutritional immunity has traditionally been understood as the active sequestration of essential trace metals (such as iron and zinc) to starve invading microorganisms, this concept has broader implications within the intestinal ecosystem.^66^ Here, it encompasses host-mediated strict restriction of macronutrients (such as free glucose) and high-potential electron acceptors (such as nitrate).^67^^,^^68^ By deliberately limiting the bioavailability of these key substrates, the host imposes significant starvation pressure on opportunistic pathogens, thereby seamlessly establishing resistance to colonization.^69^ In response, opportunistic pathogens have developed counter-strategies to exploit metabolic byproducts generated during host inflammation—such as glucose and nitrate—as privileged resources, thereby gaining a competitive edge (Figure 2).^68^

### 4.1. The glucose axis: from nutritional constraint to nutritional leakage

Beyond oxygen, glucose availability constitutes another critical metabolic force shaping the intestinal microenvironment. Under healthy conditions, intestinal epithelial cells efficiently transport glucose into the bloodstream via basolateral *GLUT2*, maintaining an “ultra-low sugar” environment within the lumen, particularly in the colon.^70^ However, high-sugar diets or hyperglycemic states disrupt this homeostasis. Intestinal sweet taste receptors, Taste receptor type 1 member (*T1R*)2, *T1R3*, detect elevated glucose concentrations, activating downstream signaling that drives the maladaptive translocation of *GLUT2*-containing vesicles to the apical membrane. While this mechanism aims to maximize energy absorption, excessive intracellular glucose flux induces transcriptional reprogramming in epithelial cells, specifically suppressing the expression of tight junction proteins Zonula Occludens-1 (*ZO-1*), *Occludin.*^71^^,^^72^

This metabolic cascade disrupts nutrient partitioning through two primary routes: unabsorbed dietary glucose spills into the distal intestine, and compromised tight junctions allow glucose-rich interstitial fluid to leak into the lumen. This “niche opening” depends on specific physiological and ecological thresholds. In the small intestine, active glucose transport via Sodium-glucose cotransporter 1 (*SGLT1*) saturates at luminal concentrations of approximately 30 mM.^73^ Sugar intake exceeding this capacity forces unabsorbed simple carbohydrates into the colon, altering the microbial competitive environment. This carbon influx suppresses obligate anaerobes dependent on complex fiber fermentation (*Prevotella*, *Lactobacillus*) and selectively enriches sugar-utilizing facultative anaerobes.^74‐76^

Dysbiosis initiates when glucose accumulation exceeds the metabolic buffering capacity of the commensal community. Upon crossing this competitive threshold, opportunistic pathogens within the phylum *Pseudomonadota* (formerly *Proteobacteria*) (such as *Salmonella* and *Escherichia coli*) rapidly consume the carbon surplus. By coupling leaked glucose with inflammation-derived electron acceptors, these bacteria bypass traditional fermentation to undergo respiratory expansion.^77^ Crucially, massive glucose inundation in the distal intestine is not required to precipitate dysbiosis; even a low millimolar accumulation (~1–5 mM) of free sugars is sufficient to disrupt the ecosystem.^77^^,^^78^ The subsequent overgrowth of Gram-negative bacteria, combined with barrier compromise, drives massive LPS translocation into the circulation, causing metabolic endotoxemia. LPS activation of Toll-like receptor 4 (*TLR4*) induces systemic inflammation and worsens insulin resistance, thereby establishing a “hyperglycemia–barrier dysfunction–dysbiosis–inflammation” cycle.^79^^,^^80^ Consequently, this carbon-driven niche shift represents a core mechanistic link between metabolic disease and intestinal dysbiosis (Figure 2).

### 4.2. Alternative electron acceptors: the inflammation-driven “respiratory niche.”

In the healthy colon, nitrate concentrations are negligible. However, inflammation triggers a dramatic surge in luminal nitrate, a key driver of *Enterobacteriaceae* expansion. This process originates from the host immune response: pro-inflammatory cytokines Interferon gamma (*IFN*)-*γ*, Tumor necrosis factor alpha (*TNF-α*) strongly induce intestinal epithelial cells to express inducible nitric oxide synthase (*iNOS*), producing nitric oxide.^81^ Within the ROS-rich inflammatory microenvironment, NO rapidly reacts to form peroxynitrite, which ultimately isomerizes into stable nitrate.^82^

This transforms the intestine from an electron-acceptor-limited environment into a nitrate-rich “respiratory niche”.^83^ Nitrate acts as a stringent ecological filter: it suppresses obligate anaerobes lacking respiratory pathways while serving as a “metabolic accelerant” for facultative anaerobes. This enables their explosive growth through respiratory dominance, even in the absence of exogenous infection.^84^^,^^85^

Furthermore, inflammation profoundly reshapes sulfur metabolism. Hydrogen sulfide (H₂S) produced by symbionts is typically detoxified by the host into thiosulfate.^86^ During inflammation, however, ROS oxidize thiosulfate into tetrathionate. This not only creates a novel electron acceptor but also establishes a sophisticated “Arsonist” strategy: pathogens like *Salmonella* actively induce host inflammation to generate tetrathionate, thereby securing an exclusive respiratory privilege under anaerobic conditions. This allows them to outcompete commensal bacteria incapable of utilizing this pathway.^87^^,^^88^ Collectively, these observations highlight that the intestinal nutrient landscape is actively sculpted by host metabolic fidelity. The pathological leakage of glucose and electron acceptors effectively bypasses nutritional immunity, transforming the luminal environment from a restrictive “fermentation bioreactor” into a permissive “respiratory niche” that favors facultative anaerobes.^25^ This underscores that colonization resistance is not merely about physical exclusion but is strictly contingent upon host metabolic homeostasis. This principle of bioenergetic gating extends beyond nutrient control to the maintenance of physical defenses—specifically, the mucus and antimicrobial peptide barriers (Figure 2).

## 5. Barrier metabolism and immune defense

The intestinal mucosal barrier functions not merely as a physical boundary but as a highly active immunometabolic interface.^89^ The host actively orchestrates microbial spatial distribution by secreting mucins and antimicrobial peptides (AMPs) via goblet cells and Paneth cells, respectively.^90^ This secretory function is not static; rather, it is dynamically regulated by host bioenergetic status, organelle homeostasis (specifically of the endoplasmic reticulum and mitochondria), and nutrient-sensing pathways.

### 5.1. Mucin metabolism: biosynthetic burden and nutrient screening

The mucus layer, anchored by *MUC2*, constitutes the host's first line of defense. As a 2.5 MDa giant glycoprotein, *MUC2* biosynthesis presents a formidable metabolic challenge: it requires not only intricate scaffold assembly within the endoplasmic reticulum (ER) but also heavy reliance on the hexosamine biosynthetic pathway (HBP), consuming substantial glucose and glutamine to maintain up to 80% O-glycosylation.^91^ This exceptionally high “metabolic synthesis load” tightly couples mucus barrier integrity to host energy status. Consequently, goblet cells are contingently dependent on the unfolded protein response (*UPR*) to maintain ER homeostasis, with the Inositol-requiring enzyme 1 beta (IRE1β)-Anterior gradient 2 **(***AGR2***)** axis functioning as the core quality-control mechanism for *MUC2* folding.^92‐94^ However, under metabolic stress (a high-fat diet), lipid overload disrupts ER membrane fluidity, leading to maladaptive *UPR* and *AGR2* retention. This results in intracellular accumulation of MUC2 precursors and a sharp decline in mature protein secretion, ultimately causing barrier thinning and increased permeability.^95^^,^^96^

Host-synthesized *MUC2* O-glycans also serve as a “privileged nutrient source,” selectively favoring beneficial symbionts. Keystone taxa such as Akkermansia muciniphila utilize mucins as their sole carbon and nitrogen sources, establishing a robust niche within the outer mucus layer.^97^^,^^98^ In turn, the metabolic activity of *A. muciniphila* stimulates goblet cell mucin secretion, establishing a positive feedback loop of “mucus secretion–bacterial growth–barrier enhancement”.^99^ However, when host metabolic dysfunction impairs *MUC2* secretion, the resulting starvation effect reduces mucus-dependent bacterial communities, such as *Akkermansia.*^100‐102^ The subsequent expansion of facultative anaerobes (*Enterobacteriaceae*) is likely a broader ecological cascade effect rather than a simple consequence of mucus depletion. The breakdown of this physical scaffold is likely to interact dynamically with other environmental factors in the intestine. For example, the absence of key mucus-degrading bacteria drastically alters the competitive landscape among local microbes, thereby effectively lifting metabolic suppression of opportunistic pathogens.^103^ Furthermore, the compromised mucus layer may be highly susceptible to dietary fluctuations; in the absence of sufficient dietary fiber, other commensal microbes may be forced to feed on host mucosal carbohydrates, thereby accelerating the degradation of an already thinned barrier.^104^ Furthermore, given that the mucus network influences host immune effector molecules such as secretory IgA (sIgA), mucosal thinning may impair intestinal immune function, thereby weakening local immune exclusion.^103^ Against this backdrop, *Enterobacteriaceae*—with their versatile metabolic mechanisms independent of mucus—appear to be exploiting this compound vulnerability. Their rapid colonization suggests that, driven by the concurrent collapse of physical, competitive, and immune constraints, the system is shifting from a “mucus-driven symbiotic mode” to an “inflammation-driven pathogenic mode”^105^^,^^106^ (Figure 2).

### 5.2. Paneth cell immunometabolism: energy-gated chemical defense

Paneth cells secrete *α*-defensins and lysozyme, forming the intestinal chemical defense barrier. Immunometabolic evidence indicates that this secretory capacity is strictly gated by cellular bioenergetics, with Mechanistic Target Of Rapamycin Kinase (*mTORC1*)acting as a critical “metabolic checkpoint”.^107^ The formation of secretory granules imposes a high protein-synthesis burden, making Paneth cells heavily reliant on ATP and biosynthetic precursors derived from glycolysis.^36^

During infection or the acute phase of IBD, proinflammatory signaling and metabolic stress induce mitochondrial structural damage. Concurrently, inflammation inhibits the UPR by downregulating key *IRE1* pathway genes, including Endoplasmic Reticulum to Nucleus Signaling (ERN)1 and *ERN2*, leading to a marked reduction in AMP secretion.^108^^,^^109^ This “chemical barrier failure” disrupts the host's ability to differentially screen the microbiota. Under physiological conditions, *α*-defensins specifically eliminate bacteria via electrostatic attraction, as cationic peptide segments bind to anionic bacterial surfaces. While core commensals (*Bacteroidota*) have evolved resistance by modifying LPS to reduce their surface negative charge, facultative anaerobic pathogens (*Salmonella*, *E. coli*) remain highly susceptible.^110^^,^^111^ Thus, when host metabolic exhaustion silences Paneth cell function, this selective inhibitory pressure is lost, allowing previously constrained facultative anaerobes to proliferate unchecked.^112^ This “immune brake failure,” combined with oxygen leakage and nitrate accumulation, collectively drives pathological dysbiosis. Collectively, these findings highlight that the mucosal barrier is not a static physical shield but an energetically expensive defense system contingent upon host metabolic health. The breakdown of these “metabolic checkpoints”—whether via ER stress in goblet cells or mitochondrial dysfunction in Paneth cells—compromises the host's selective filtration capacity, creating a permissive niche for facultative anaerobes. This mechanistic insight implies that restoring ecological balance transcends simple antimicrobial suppression; it requires the bioenergetic rehabilitation of the host. Accordingly, the subsequent section examines therapeutic strategies to recalibrate these critical metabolic regulators (Figure 2).

## 6. The vicious cycle between host metabolism and pathogenic microbiota

The transition from intestinal homeostasis to persistent dysbiosis represents a systemic collapse of host ecological governance. As established in the preceding sections, the host's metabolic capacity to enforce a hypoxic, nutrient-restricted environment ultimately dictates whether the microbiota defaults to symbiotic fermentation or exploits pathogenic respiration. The collapse of this top-down control is rooted in a central physiological pivot: host mitochondrial bioenergetic failure. This breakdown is not a downstream consequence of microbial imbalance, but the direct mechanistic driver that forces the respiration-fermentation switch within the intestinal epithelium, subsequently dismantling the entire mucosal defense architecture.

Under healthy conditions, obligate anaerobes produce SCFA, particularly butyrate, through the fermentation of dietary fiber. As previously noted, butyrate serves not only as the primary energy source for colonic epithelium but also maintains efficient mitochondrial oxidative phosphorylation by activating the nuclear receptor *PPAR-γ*, thereby preserving the hypoxic environment within the intestines.^113^^,^^114^ However, this pathogenic cycle initiates when inflammatory insults induce the production of excess nitric oxide and ROS. At the molecular level, NO competitively binds to cytochrome c oxidase (Complex IV), while ROS destroy the iron-sulfur clusters of critical tricarboxylic acid (TCA) cycle enzymes like aconitase.^115^ This physical paralysis of the electron transport chain triggers a severe intracellular ATP deficit. To survive this profound bioenergetic crisis, colonocytes are forced into a compensatory metabolic shift from efficient oxidative phosphorylation (respiration) to *HIF-1α*-mediated anaerobic glycolysis (fermentation) (Figure 2).^41^

This forced metabolic reprogramming triggers a synchronized collapse of the host's ecological filters. First, the cessation of mitochondrial *β*-oxidation abruptly abolishes the epithelial oxygen sink, causing unconsumed oxygen to leak into the lumen, while inflammation and oxidative stress simultaneously drive nitrate accumulation.^116^ Second, the profound ATP starvation directly impairs highly energy-demanding barrier functions. It triggers severe endoplasmic reticulum (ER) stress that halts *MUC2* synthesis in goblet cells^117^ and concurrently stifles the secretion of antimicrobial peptides (AMPs) by Paneth cells.^108^^,^^109^

Consequently, the intestinal microenvironment is fundamentally altered. The breakdown of spatial and chemical segregation, combined with the influx of leaked oxygen and nitrate, effectively eradicates the competitive advantage of obligate anaerobes. Facultative anaerobes, such as *Enterobacteriaceae*, exploit these abundant electron acceptors to bypass the fermentation bottleneck, initiating an explosive respiratory expansion.^118^^,^^119^ In a destructive feedback loop, these overgrown pathogens release massive quantities of LPS and virulence factors, persistently activating the host *TLR4/Myeloid differentiation primary response gene 88 (MyD88)/Nuclear factor kappa B (NF-κB)* pathway.^120^^,^^121^ This sustained inflammation continuously fuels NO and ROS production,^122^^,^^123^ permanently locking the host epithelium in a state of bioenergetic failure.

**Figure 3.**
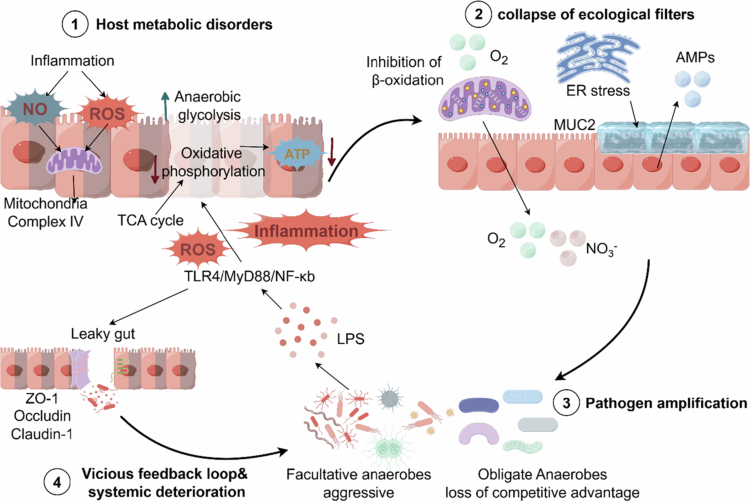
The vicious cycle of host metabolic dysregulation and pathogenic microbial communities. Inflammatory stimuli initiate this cycle by generating excessive nitric oxide (NO) and reactive oxygen species (ROS); the former and latter exert their effects by competitively inhibiting cytochrome c oxidase (complex IV) and damaging key enzymes in the citric acid cycle (such as succinate dehydrogenase), respectively. Disruption of the electron transport chain triggers a severe intracellular ATP shortage, forcing colonic cells to shift from efficient oxidative phosphorylation to anaerobic glycolysis. This forced metabolic reprogramming subsequently disrupts mucosal defenses: the cessation of mitochondrial *β*-oxidation eliminates epithelial oxygen deposition, leading to the leakage of unconsumed oxygen and nitrates into the lumen, while severe ATP depletion triggers severe endoplasmic reticulum stress, thereby halting the highly energy-intensive synthesis of the mucus layer (MUC2) and antimicrobial peptides (AMPs). Consequently, the influx of these high-potential electron acceptors into the lumen, coupled with the weakening of physical and chemical barriers, completely eliminates the competitive advantage of obligate anaerobes. This opening of the ecological niche allows facultative anaerobes (particularly Enterobacteriaceae) to rapidly utilize leaked substrates, triggering an explosive expansion of respiration. In a vicious cycle, these proliferating opportunistic pathogens release large amounts of lipopolysaccharide (LPS), continuously activating the host’s Toll-like receptor 4 (*TLR4*)/Myeloid differentiation primary response gene 88 (*MyD88*)/Nuclear factor kappa B (*NF-κB*) signaling pathway, thereby constantly promoting the production of nitric oxide (NO) and reactive oxygen species (ROS), which in turn permanently plunges the epithelial tissue into bioenergetic failure. Ultimately, this persistent energy crisis disrupts the assembly of tight junction proteins Zonula Occludens-1 (*ZO-1*), *Occludin*, and *Claudin-1*, leading to increased intestinal permeability and triggering chronic systemic endotoxemia. Created in Figdraw.com.

Ultimately, the energy crisis directly disrupts the assembly of tight junction proteins (*ZO-1*, *Occludin*, *Claudin-1*), increasing intestinal permeability.^124^ This leaky intestinal state facilitates the massive entry of intestinal microbiota and their metabolites into the systemic circulation, triggering chronic systemic endotoxemia. By this stage, the initial localized metabolic disturbance has evolved into a systemic pathological state, establishing the irreversibility of this vicious cycle (Figure 3). This integrated paradigm explains why simple microbial replenishment strategies often struggle to restore long-term homeostasis, underscoring the urgent need for novel interventions that break the cycle by restoring host bioenergetics.

## 7. Intervention strategies: targeting host metabolism to reshape the intestinal microbiota

**Figure 4.**
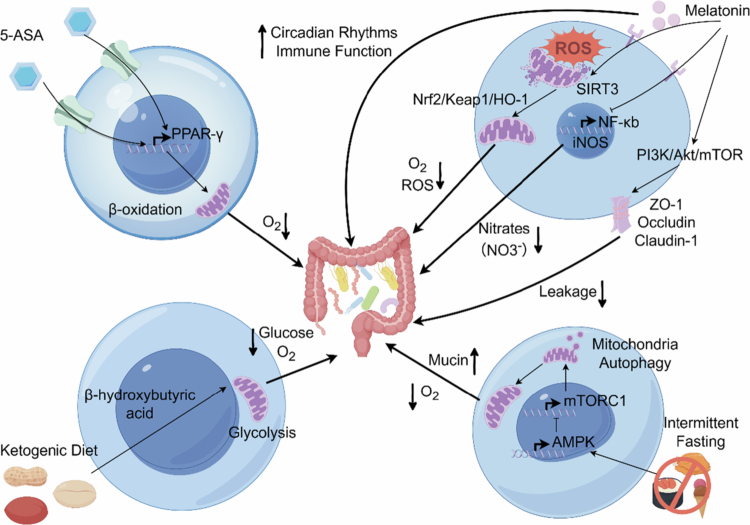
Intervention strategies targeting host metabolism. 1. Nuclear transcription reprogramming (top left): 5-Aminosalicylic acid (5-ASA), acting as a *PPAR-γ* agonist, reverses metabolic reprogramming in epithelial cells, restores mitochondrial beta-oxidation and intestinal hypoxia, thereby inhibiting respiration of facultative anaerobic bacteria. 2. Mitochondrial protection and antioxidant defense (top right): Melatonin clears mitochondrial ROS by activating the Sirtuin 3 (*SIRT3*)/Nuclear respiratory factor (*NRF*)2 axis, safeguards electron transport chain integrity, and suppresses Nuclear factor kappa B (*NF-κB*) expression to reduce inflammation-associated nitrate production. Additionally, it protects the intestinal barrier by activating Phosphoinositide 3-kinase (*PI3K*)/Protein kinase B (Akt)/Mechanistic target of rapamycin (*mTOR*), preventing pathogen translocation, and safeguarding host intestinal rhythmicity and immune function. 3. Metabolic Substrate Conversion and Quality Control (Bottom): The ketogenic diet (KD) provides ketone bodies (*β*-HB) as an alternative fuel, bypassing impaired glycolysis to restore aerobic metabolism. Intermittent fasting (IF) induces mitochondrial autophagy by activating AMP-activated protein kinase (*AMPK*), specifically eliminating dysfunctional “leaky” mitochondria. Created in Figdraw.com.

Historically, therapies for dysbiosis have followed a “microbe-centric” approach, such as probiotic supplementation and fecal microbiota transplantation (FMT). However, clinical outcomes reveal that exogenous strains often fail to engraft long-term because these strategies neglect the host's pathological environment, which acts as a barrier to colonization.^125^^,^^126^ Emerging research is shifting toward a “host-centric” paradigm, positing that host metabolic states—particularly mitochondrial function and intestinal oxygen consumption—are the fundamental determinants of microbial community structure.^32^ The leakage of oxygen and nitrate, driven by host metabolic dysregulation, is the primary force fueling the expansion of facultative anaerobes (*Enterobacteriaceae*).^85^ Therefore, we propose a novel intervention framework: restoring host metabolic homeostasis to reconstruct the physiological hypoxic niche. This strategy aims to "starve" pathogenic symbionts by re-imposing ecological restrictions, thereby fundamentally reestablishing microecological balance (Figure 4).

### 7.1. Targeting host mitochondrial function restores intestinal oxygen consumption

The primary strategy to break the vicious cycle in the intestine is to restore the oxygen consumption capacity of damaged epithelial cells, thereby depriving facultative anaerobic bacteria of their survival niche by reactivating aerobic metabolism. As previously discussed, diminished mitochondrial *β*-oxidation efficiency is the root cause of oxygen leakage into the intestinal lumen, a process meticulously regulated by the nuclear transcription factor *PPAR-γ*. Therefore, identifying interventions capable of reshaping the epithelial cell transcriptional profile and compelling its return to oxidative phosphorylation becomes pivotal for restoring physiological hypoxia. *PPAR-**γ*** is the master transcription factor governing mitochondrial *β*-oxidation in the colonic epithelium. In IBD, *PPAR-**γ*** expression is commonly downregulated, shifting epithelial metabolism from efficient *β*-oxidation to inefficient glycolysis and triggering luminal oxygen leakage. 5-Aminosalicylic acid (5-ASA/Mesalamine), a first-line IBD therapy traditionally viewed as an anti-inflammatory agent, has been mechanistically redefined as a “host metabolic regulator.”5-ASA directly activates epithelial *PPAR-γ*, reactivating butyrate oxidation and restoring mitochondrial bioenergetics. Crucially, this metabolic reprogramming re-establishes intestinal hypoxia,^127^^,^^128^ directly depriving pathogens like *E. coli* of the aerobic respiration they require. Compellingly, 5-ASA loses both its microbiota-modulating and anti-inflammatory efficacy in mice with epithelium-specific *PPAR-**γ*** deletion,^129^ demonstrating that its therapeutic success is strictly contingent on the integrity of host metabolism (Figure 4).

### 7.2. Restricting the respiratory electron acceptors of pathogenic bacteria

Beyond controlling oxygen leakage, blocking respiratory electron acceptors (such as nitrates) generated by inflammation-driven pathways represents another core strategy to limit the expansion of pathogenic symbionts. During inflammatory states, the surge of *iNOS* not only fuels bacterial growth but also damages mitochondria through NO. This implies that effective interventions must possess dual properties: protecting mitochondria from damage through antioxidant defenses while simultaneously suppressing inflammatory pathways to disrupt abnormal energy supply. Against this backdrop, an endogenous molecule with both circadian regulation and potent antioxidant properties has demonstrated significant therapeutic potential. While traditionally viewed as a pineal hormone regulating circadian rhythms, the gastrointestinal tract is actually the body's largest reservoir of melatonin (with concentrations 10–100 times higher than in blood), synthesized primarily by enterochromaffin cells. Here, it functions as a potent paracrine modulator of the intestinal microenvironment.^130^

Melatonin targets mitochondria, whose dysfunction initiates oxygen leakage. By upregulating the mitochondrial deacetylase Sirtuin 3 (*SIRT3*), melatonin activates antioxidant enzymes (*SOD2*) and the Kelch-like ECH-associated protein 1 (*Keap1*)/*Nrf2* pathway.^131^^,^^132^ This dual antioxidant mechanism preserves electron transport chain integrity, ensuring efficient epithelial oxygen consumption and maintaining luminal anaerobiosis. Furthermore, melatonin inhibits *NF-κB* signaling to downregulate *iNOS*, thereby cutting off the inflammation-driven nitrate supply.^133^ Deprived of both oxygen and nitrate, *Enterobacteriaceae* lose their respiratory growth advantage. In mouse models of metabolic dysfunction and intestinal inflammation, exogenous melatonin supplementation consistently results in a direct remodeling of the intestinal microbiome, characterized by a significant reduction in the relative abundance of *Pseudomonadota* (a phylum that includes *Enterobacteriaceae*) and a corresponding recovery of SCFA-producing obligate anaerobes.^134^^,^^135^ Crucially, recent in vivo evidence confirms that melatonin drives this microbial shift not through direct antimicrobial action, but by mitigating stress-induced mucosal damage and repairing the host microenvironment.^136^

Additionally, melatonin reverses stress-induced *MUC2* downregulation by inhibiting ROS-mediated promoter methylation and repairing tight junctions via the Phosphoinositide 3-kinase (*PI3K*)/Protein kinase B (*Akt*)/Mechanistic target of rapamycin (*mTOR*) pathway.^137^^,^^138^ Given that sleep deprivation itself induces dysbiosis, melatonin improves the microbiome not only as a supplement but also by realigning host circadian rhythms, restoring the periodicity of intestinal motility and immune secretion^11^ (Figure 4).

### 7.3. Improve microbial metabolic substrates

When the intestinal ecosystem enters a state of depletion of butyrate-producing bacteria and epithelial energy starvation, relying solely on restoring endogenous metabolism often fails to yield rapid results.^139^ At this juncture, strategically altering the microbiota's metabolic substrates through external interventions is a key approach to reshaping the microenvironment. This provides energy to epithelial cells while simultaneously imposing nutritional pressure on carbohydrate-dependent pathogens. By precisely adjusting dietary patterns, we can forcibly restart the damaged metabolic infrastructure from two dimensions: cellular energy supply and nutritional immunity.^68^ The Ketogenic Diet (KD), a high-fat, low-carbohydrate regimen traditionally used for neurological disorders, profoundly reshapes the intestinal microbiome by shifting the host's energy substrate.^140^ For example, mouse models fed a ketogenic diet exhibit rapid and reproducible changes in intestinal microbiota composition, a process directly driven by the production of host ketone bodies.^141^ The mechanism involves two aspects: restoring oxygen levels in epithelial cells and depriving pathogens of nutrients.

Under KD, liver-derived *β*-hydroxybutyrate (*β*-HB) serves as a critical alternative fuel for the colonic epithelium. The oxidative metabolism of *β*-HB enhances mitochondrial complex I stability and ATP synthesis,^142^ compelling epithelial cells to resume high rates of oxygen consumption and thus restoring the hypoxic barrier.^143^ Concurrently, the drastic restriction of carbohydrates imposes “starvation pressure” on monosaccharide-dependent pathogens (*E. coli*, *Salmonella*).^144^ However, the efficacy of KD is context-dependent: its microbiological safety critically relies on the fat source. KDs based on animal-derived saturated fats (lard) promote taurocholate secretion, enriching the sulfur-reducing bacterium *Bilophila wadsworthia* and compromising the barrier. Conversely, plant-derived unsaturated fats appear to mitigate this risk.^145^

Intermittent Fasting (IF) transcends simple calorie restriction; it functions by creating windows of energy deprivation that trigger ancient cellular repair mechanisms. Fasting elevates the cellular AMP/ATP ratio, activating the energy sensor AMP-activated protein kinase (*AMPK*) and relieving *mTORC1* inhibition.^146^ This initiates mitophagy—the selective clearance of inflammation-damaged, “leaky” mitochondria—ensuring that the mitochondrial pool remains highly efficient at consuming oxygen.^147^ Furthermore, fasting selects for mucus-adapted commensals, such as Akkermansia, and shifts the microbiota toward an anti-inflammatory phenotype.^148^^,^^149^ Collectively, the therapeutic efficacy of these diverse strategies—spanning pharmacotherapies to dietary regimens—underscores the validity of the “host metabolic regulation” paradigm. Although targeting distinct nodes of cellular bioenergetics, these interventions converge on a unified ecological outcome: the restoration of the physiological hypoxia barrier and the reinforcement of nutritional immunity. This transition from a microbe-centric to a host-centric perspective implies that resolving dysbiosis necessitates more than the mere introduction of beneficial strains; rather, it demands the reconstruction of the metabolic infrastructure that sustains them. Consequently, future clinical translation must prioritize the precise modulation of host mitochondrial function and fuel utilization, effectively conditioning the intestinal “metabolic soil” to foster a resilient microbial ecosystem (Figure 4) (Table 1).

**Table 1.** Host metabolism and intestinal microbiota composition classification.

Metabolic pattern	Homeostatic Mechanism	Pathological mechanisms	Pathological microbiota	Reference
**1. Oxygen gradient**	Mitochondrial beta-oxidation	Inflammation, mitochondrial damage, glycolysis	*Enterobacteriaceae* *Escherichia coli* *Parastaphylococcus Haemophilus parainfluenzae*	[63,64]
**2. Electron receptor**	Symbiotic microbiota degrade nitrate	Oxidative stress, Inflammation	*Salmonella*, *Escherichia coli*	[84,85]
**3. Nutritional immunity**	Lipocalin-2 isolates iron, *GLUT2* polar distribution restricts glucose	Hyperglycemia, inflammation, leaky intestine	*Salmonella* *Escherichia coli* *Listeria monocytogenes*	[75,76]
**4. Barrier metabolism**	Endoplasmic reticulum, Paneth cells efficiently synthesize *MUC2*, AMPs	Mitochondrial dysfunctionReticulum stress	*Salmonella* *Escherichia coli*	[105,112]

## 8. Conclusion

In various inflammatory pathologies, intestinal dysbiosis is not a random fluctuation in microbial populations, but rather a direct consequence of the collapse of host-driven ecological filtering mechanisms. This review integrates a “top-down” paradigm, demonstrating that host epithelial metabolism is the ultimate regulator of the luminal microenvironment. Under steady-state conditions, colonic epithelial cells utilize butyrate produced by the microbiota to sustain robust mitochondrial *β*-oxidation—a core metabolic process that actively consumes local oxygen, limits the availability of electron acceptors in the respiratory chain, and generates the ATP required to maintain the mucosal barrier. However, when inflammatory injury forces epithelial cells into bioenergetic failure and a shift to anaerobic glycolysis, this regulatory mechanism systematically breaks down. The cessation of *β*-oxidation eliminates the oxygen consumption pool, leading to the leakage of oxygen and nitrates into the lumen, while the concurrent energy crisis disrupts the maintenance of physical and chemical defense mechanisms. Opportunistic pathogens (such as *Enterobacteriaceae*) actively exploit these newly available electron acceptors, bypassing strict fermentation bottlenecks and triggering an explosive expansion of respiration. By mapping this comprehensive metabolic pathway, it becomes clear that purely microbe-centric therapies are inherently unable to overcome the pathological host environment. Achieving long-term ecological homeostasis requires a paradigm shift: future interventions must strategically target recalibrating host epithelial cell bioenergetics to fundamentally restore the damaged metabolic soil.
